# A multifaceted provider-centred intervention versus usual care to improve the recognition and diagnosis of depression in primary health care: a hybrid study

**DOI:** 10.1017/S1463423623000300

**Published:** 2023-07-10

**Authors:** Eva Vanesa Nogueras, Nazaret Cantero, María Macías, José Miguel Morales-Asencio, José María García-Herrera Pérez-Bryan, María M. Hurtado

**Affiliations:** 1 Instituto de Investigación Biomédica de Málaga (IBIMA), Málaga, Spain; 2 Mental Health Unit, Regional University Hospital, Málaga, Spain; 3 Faculty of Health Sciences, Universidad de Málaga, Málaga, Spain

**Keywords:** depression, diagnostic tests (PHQ-9), mental health, mood disorder, practice management, primary care, psychiatry

## Abstract

**Background:**

The aim of this study was to evaluate the impact of a multifaceted intervention to implement an adapted guideline for the management of depression in primary health care.

**Methods:**

A hybrid trial was carried out to determine the effect of a multicomponent provider-centred intervention to improve the detection and diagnosis of depression in primary care, as part of the guideline implementation process, and to collect information about barriers and facilitators in a real-world context. Before the multicomponent intervention, a descriptive cross-sectional study was performed to assess the population prevalence of depression in the participating health centres and to detect possible differences. Subsequently, a quasi-experimental two-phase study was carried out with a concurrent control group to assess the impact of the multicomponent intervention on the main outcomes (detection of depression, evaluation of its severity and the use of structured methods to support the diagnosis).

**Results:**

Nine-hundred seventy-four patients took part in the first phase. According to their clinical records, the prevalence of depression ranged from 7.2% to 7.9%, and there were no significant differences between the health centres scheduled to receive the intervention and those in the control group. In the experimental phase, 797 randomly selected participants received the multicomponent intervention. Adjusted multivariable analysis performed before the implementation revealed no significant differences in depression between the experimental and control groups. However, after the intervention, modest but significant differences were observed, which persisted at 1 year after the intervention.

**Conclusions:**

A multicomponent intervention for the implementation of a clinical guideline for the management of depression in primary care produced improvements in the identification of depression and in the degree of severity recorded.

## Background

In countries such as Spain where primary health care plays a gatekeeper role, patients with depression usually have their first contact with health care in this setting (Aragonès *et al.*, 2017). In fact, nearly 80% of antidepressant prescriptions are written by practitioners who are not mental healthcare providers (Mark *et al.*, 2009), and most of these are in primary care. In this context, researchers have identified cases in which non-depressed patients have been diagnosed with depression (Aragonès *et al.*, 2006). The opposite phenomenon (patients who have depression but are not diagnosed) has also been reported (Mitchell *et al.*, 2009). Moreover, the diagnosis of depression is often not recorded in the patient’s clinical history (Mitchell *et al.*, 2009). Even more concerning is the fact that many people with no depression are treated with antidepressants. In the case of older people, almost half of those treated do not meet criteria that justify the diagnosis (Maust *et al.*, 2017).

Researchers have long been aware of an important gap between clinical knowledge and practice in the management of depression (Wainberg *et al.*, 2017), and clinical guidelines to improve decision making in depression care have been proposed to address this issue (Nogueras *et al.*, 2017). Some studies have reported significant improvements in depression care when barriers are addressed effectively and clinical guidelines are followed (Hepner *et al.*, 2007). Provider-centred interventions in primary care settings reveal some improvements in the quality of medication prescribing, with a probable dose–response relation between the intensity of the intervention and the effect size (Pedersen *et al.*, 2018). In other words, multicomponent interventions seem to have more impact on the treatment recommended by clinical guidelines than interventions that include only an informational or educational component, as different forms of guideline distribution or a brief presentation of the topic (Pedersen *et al.*, 2018). However, there are few studies on the impact of provider-centred intervention on the detection, evaluation and diagnosis process.

The general aim of the present study was to evaluate the impact of a multifaceted provider-centred intervention to improve the detection and diagnosis of depression in primary health care. The specific aims were to assess the impact of this intervention on the detection of depressive disorders by primary care practitioners (PCPs), their ability to classify the severity of the depressive disorder and their use of standardised instruments to support the diagnosis, as recommended in national clinical guidelines (García-Herrera Pérez-Bryan *et al.*, 2011).

## Materials and methods

Implementation of the clinical guidelines was evaluated using a hybrid type 1 approach (Curran *et al.*, 2012) to test the effect of a multifaceted provider-centred intervention and to collect information during the implementation process about barriers and facilitators in a real-world context. The results of the phase for detecting barriers and facilitators have been extensively reported elsewhere (Nogueras *et al.*, 2017).

To evaluate the implementation, a three-phase study was carried out. The first phase, prior to the implementation of the clinical guidelines, was conducted through a descriptive cross-sectional study to assess the population prevalence of depression in the participating health centres. The aim of this phase was to verify possible differences due to socioeconomic factors. This issue, which has been widely reported in the literature (Araya *et al.*, 2018; Patel *et al.*, 2018), could act as a confounder in the interpretation of the results of the implementation process. This a priori exploration was indicated, moreover, because the study covered a wide geographical area that included urban districts with diverse income and education levels.

The second, quasi-experimental, phase involved patients who had been prescribed antidepressants (and thus were more likely to have a depressive disorder) to determine the main outcomes (detection of depression, evaluation of the severity of depression and the use of structured methods to support the diagnosis) at baseline, prior to the implementation of the clinical guidelines. In the third phase, after its implementation, these outcomes were evaluated in a new sample of patients, currently taking antidepressants.

The study was conducted in the Malaga Primary Health Care District (Spain), in which 12 of the 32 health centres are assigned to the two Community Mental Health Services (CMHS) of the Regional University Hospital of Malaga. These CMHS, via 170 PCPs, attend a population of 251 259, which was the reference population for phase one of this study.

For phases two and three, aimed at evaluating the impact of a multifaceted provider-centred intervention on the detection and diagnosis of depression in primary care, two samples of patients were selected from those belonging to the participating health centres who had been prescribed any antidepressant drug in the period 2011–2014. These patients were the most likely to have a diagnosis of depression, even if no such diagnosis was recorded in the clinical records (indeed, one of the aims of the clinical guidelines is to raise the visibility of this non-registered population). The data for prescriptions were provided by the pharmacy service of the Primary Health Care District.

One of the CMHS (with its corresponding health centres) was selected as the intervention group, and the other one as the control group. The health centres could not be cluster randomised due to organisational issues. The main barrier to this study was the fact that the research team was employed in one of the CMHS and did not have access to the other area to implement the clinical guidelines. For this reason, the second area was taken as the control group. Nevertheless, the individual selection of participants at each health centre was randomised (by a computerised system) from the complete list of patients who had been prescribed antidepressants and met the inclusion criteria.

The inclusion criteria for the first phase were that patients should form part of the population census of the participating health centres, be at least 18 years of age and have made at least one visit to the health centre for any reason during the year prior to the study. Patients with a previous diagnosis of depression were excluded.

For the second and third phases, the study population was composed of patients aged 18 years or older, prescribed an antidepressant and being treated in one of the health centres. Patients were excluded if their residence in the health district was only temporary, if the antidepressant prescription was for purposes other than the treatment of depression (such as other psychiatric processes, neuralgia or sleep disorders) or if they had previously been attended by Mental Health Services. Any patients lacking the cognitive or language ability to complete PHQ-9 or admitted to nursing homes or receiving home care were also excluded.

For the first phase, assuming a prevalence of 14% of major depression in the primary health care population (Caballero *et al.*, 2008), for a reference population of 251 259 people, with an accuracy of 2.5%, it was necessary to recruit 738 eligible patients. This sample size was increased by 25% to allow for possible losses, making a total initial sample of 922 patients. Stratified random sampling was conducted according to the proportion of the population assigned to each health centre and to the male:female population distribution.

For the second and third phases of the study, to detect a 15% difference in the frequency of detection of depression in the PCP’s medical records (Sinnema *et al.*, 2011, 2015), assuming an alpha value of 5% and a statistical power of 80%, we calculated that 173 participants in each group were required. This sample size was increased by 10% to allow for possible losses. The total sample size needed, thus, were 380 patients, 190 per group.

Consequently, 380 patients were selected before the implementation of the clinical guidelines. These patients were followed up for six months. Once the clinical guidelines were implemented, another 380 different patients were selected and followed for 12 months, to determine the long-term effect of the intervention. In this second phase, the sampling of patients was also randomised and stratified by centre and gender. Moreover, the research team decided during the study to increase the sample up to 410 per group, to assure statistical power.

### Development of the guideline and implementation of a multifaceted provider-centred intervention

The intervention was designed in accordance with the EPOC Taxonomy (EPOC Taxonomy, 2015). The intervention was designed and applied taking into account the Theory of Planned Behaviour (TPB) (Ajzen, 1991). Its implementability was evaluated with the Guideline Implementability Appraisal tool (Shiffman *et al.*, 2005), applied by two independent external reviewers (a PCP with special interest in mental health issues and a psychiatrist).

The components of the intervention regarding leadership, dissemination, training, support, auditing and organisational actions, and their relation with the TPB are detailed in Supplementary File 1.

In the control group health centres, patient care was provided as usual. This attention consists of detecting depression (usually on demand from the patient and without using standardised instruments or recording the severity of the symptoms) and prescribing antidepressants according to established criteria and referral to the CMHS if appropriate. When the patient is under CMHS follow-up, the treatment is reviewed and, in some cases, the patient is referred back to the primary health care.

### Data collection

Study data were collected by reviewing each PCP’s electronic medical record. For the first phase, after the random sampling, the medical records were reviewed by means of a structured sequence using a standardised form (Supplementary File 2) by trained reviewers to identify any diagnosis of depression in the last year.

In the second phase of the study, after the randomised selection of participants meeting the inclusion criteria for each participating health centre, medical records were checked at baseline and at 3 and 6 months to detect the identification of depression in clinical records, the severity of the depression and the use of PHQ-9 (an instrument that has been validated for use with the primary care population in Spain) (Muñoz-Navarro *et al.*, 2017). Together with the main outcome variables, the patients’ age, gender and comorbidities were also recorded.

**Figure 1.**
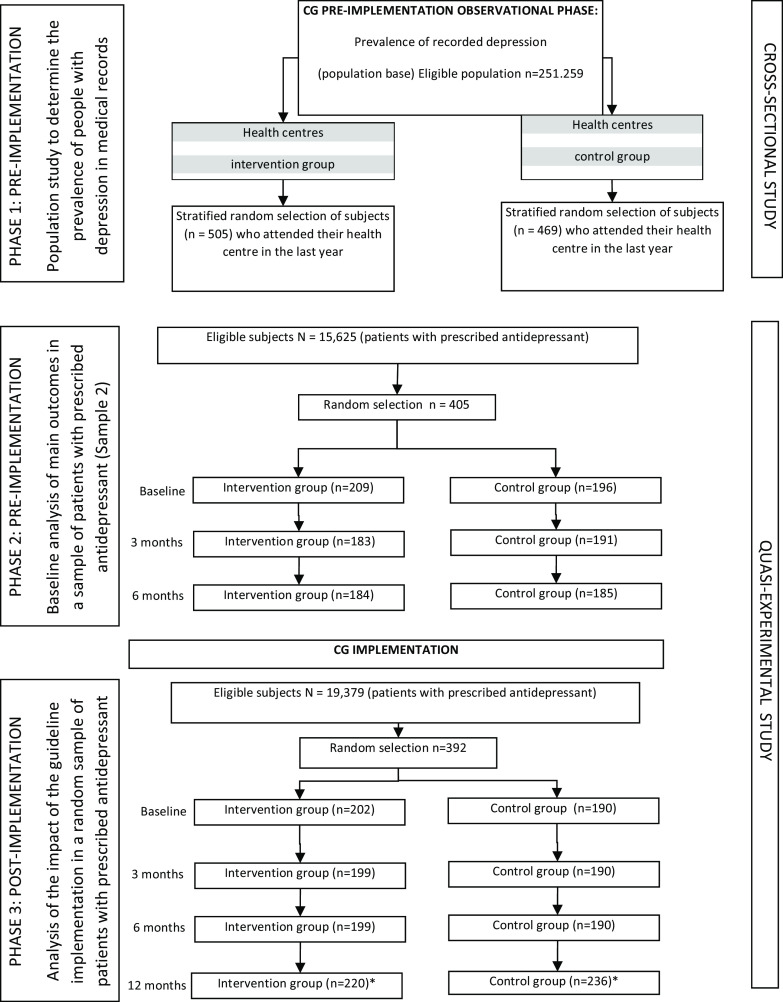
Flowchart. *Note: More subjects were recruited to replace dropouts. CG = clinical guidelines.

Once this phase had been completed, the multicomponent intervention was implemented and another sample of patients was randomly selected to evaluate the same outcome variables, at baseline and at 3, 6 and 12 months. A flowchart of the study is detailed in Fig. 1.

### Analysis

An exploratory analysis was performed to obtain descriptive statistics of the variables. The normality of distributions was evaluated by the Kolmogorov–Smirnov test.

Bivariate analyses were performed using Student’s t test or the chi-square test, according to the characteristics of the variables analysed. A multivariable analysis was carried out by logistic regression to assess the effect on the detection of depression, the recording of its severity and the use of PHQ-9, adjusted for gender and the presence of physical comorbidities, due to the high prevalence of this situation in people with depressive disorder (Kampling *et al.*, 2021). Crude and adjusted odds ratios with their respective 95% confidence intervals were calculated to estimate the impact produced. Since PCPs belonged to their respective healthcare centres, the potential clustering effect was evaluated. For this purpose, aggregate analyses were performed at the cluster level (healthcare centre) and individual analysis at the subject level, to see the differences due to the effect of aggregation by clusters. For this purpose, a multilevel logistic regression was carried out, where the dependent variables were the recording of depression in the clinical history, the stratification of the level of severity and the use of PHQ-9, and as a grouping factor of fixed effects, the variable “cluster health centre”. To estimate the variance of the multilevel models, the intraclass correlation coefficient (ICC) adjusted for logistic models was calculated. In addition, the median odds ratio (MOR) was calculated, which expresses the increase in the median risk that an individual would have if they changed their reference group for another.

All analyses were performed using SSPS 25 and Stata 15 statistical software.

## Results

The first phase of the study involved 974 participants (505 in the intervention group and 469 in the control group), selected from a total eligible population of 251 259. Of these, 472 (48.5%) were male and 502 (51.5%) female. By average age, there were no significant differences between male and female patients in the control group (male: 46.60, SD: 16.75 years versus female: 48.69, SD: 18.48 years; *t* = −1.28; *P* = 0.200) or in the intervention group (male 47.93, SD: 16.05 years versus female 50.77, SD: 19.07 years; *t* = −1.81, *P* = 0.071). Neither there were significant differences in the gender distribution between the two groups, with 51.8% of women in the control group versus 51.3% in the intervention group (χ^2^:0.027; *P* = 0.460).

Bivariate analysis of the PCPs’ clinical records revealed no difference between the intervention and control groups with respect to the population prevalence of depression (*P* = 0.718), OR = 0.91 (95% CI 0.56 to 1.46), which ranged from 7.2% to 7.9% of the 974 participants. The real precision obtained from these results, with regard to the anticipated calculation (2.5%), was 1.7%.

The second phase of the study included 861 patients, who presented no significant differences in age or physical comorbidities (Table 1). In the pre-implementation sample, there was a greater presence of men in the intervention group versus the control group (28.2% versus 18.4%, *P* = 0.025). No such difference was observed in the post-implementation sample.

**Table 1.** Baseline characteristics of the sample in the phases 2 and 3 of the study

	Intervention	Control	***p***
	n (%) and Mean (SD)	n (%) and Mean (SD)	
**Gender (Male)**			
Pre (intervention *n* = 209; control *n* = 196)	59 (28.2%)	36 (18.4%)	**0.025**
Post (intervention *n* = 220; control=236)	49 (22.3%)	62 (26.3%)	0.188
**Age**			
Pre (intervention *n* = 209; control *n* = 196)	62.30 (15.30)	62.03 (16.14)	0.867
Post (intervention *n* = 202; control = 190)	60.65 (15.36)	59.58 (16.99)	0.513
**Presence of physical comorbidities**			
Pre (intervention *n* = 209; control *n* = 196)	124 (59.3%)	108 (55.1%)	0.422
Post (intervention *n* = 220; control = 236)	129 (58.6%)	145 (61.4%)	0.303

Regarding the impact of the implementation of the clinical guidelines on the identification of depression in the clinical records, the crude analysis revealed no significant differences between the intervention group versus the control group in the pre-implementation phase, except in the baseline period (Table 2). Thus, an absolute risk reduction of 14.7 percentage points was obtained (95% CI 6.24 to 23.3, NNT 0 to 7). These findings fulfilled the anticipated statistical power.

**Table 2.** Differences in the identification of depression, level severity and use of PHQ-9, by groups

					Adjusted OR (95% CI)		
Follow-up		Intervention n (%)	Control n (%)	Crude OR (95%CI)	Group (intervention)	Gender (male)	Physical comorbidities
**Identification of depression**							
PRE	Baseline n (intervention/control) = 209/196	103 (49.3)	77 (39.3)	1.50 (1.5 to 2.2)^*^	1.5 (1 to 2.2)^*^	0.9 (0.6 to 1.4)	1.4 (1 to 2.1)
	3 months n (intervention/control) = 183/191	66 (36.1)	81 (42.4)	0.77 (0.51 to 1.2)	0.8 (0.5 to 1.2)	0.8 (0.5 to 1.4)	1.8 (1.2 to 2.8)^*^
	6 months n (intervention/control) = 184/185	65 (35.3)	82 (44.3)	0.69 (0.45 to 1.1)	0.7 (0.5 to 1.1)	0.9 (0.5 to 1.5)	1.7 (1.1 to 2.7)^*^
POST	Baseline n (intervention/control) = 202/190	140 (69.3)	109 (57.4)	1.68 (1.11 to 2.5)^*^	1.7 (1.1 to 2.5)^*^	0.5 (0.3 to 0.8)^*^	0.9 (0.6 to 1.4)
	3 months n (intervention/control) = 199/190	142 (71.4)	112 (58.9)	1.73 (1.14 to 2.6)^*^	1.7 (1.1 to 2.6)^*^	0.6 (0.4 to 0.9)^*^	0.8 (0.5 to 1.2)
	6 months n (intervention/control) = 199 /190	142 (71.4)	113 (59.5)	1.70 (1.11 to 2.6)^*^	1.7 (1.1 to 2.6)^*^	0.5 (0.3 to 0.9)^*^	0.8 (0.5 to 1.3)
	12 months n (intervention/control) = 220/236	163 (74.1)	140 (59.3)	1.96 (1.32 to 2.9)^**^	1.9 (1.3 to 2.9)^**^	0.5 (0.3 to 0.8)^*^	0.8 (0.5 to 1.2)
**Identification of the level of severity of depression**							
PRE	Baseline n (intervention/control) = 209/196	10 (4.8%)	2 (1%)	4.87 (4.87 to 22.5)^*^	5.1 (1.1 to 23.9)^*^	0.5 (0.1 to 2.5)	1 (0.3 to 3.4)
	3 months n (intervention/control) = 183/191	7 (3.8%)	0 (0%)	–	–	–	5.3 (0.6 to 45.7)
	6 months n (intervention/control) = 184/185	8 (4.3%)	0 (0%)	–	–	0.3 (0 to 2.5)	1.6 (0.4 to 6.8)
POST	Baseline n (intervention/control) = 202/190	39 (19.3%)	14 (7.4%)	3.01 (1.58 to 5.7)^**^	3 (1.6 to 5.8)^**^	1.5 (0.8 to 2.9)	0.5 (0.3 to 1)^*^
	3 months n (intervention/control) = 199/190	42 (21.1%)	14 (7.4%)	3.36 (1.77 to 6.4)^**^	3.4 (1.8 to 6.5)^**^	1.7 (0.9 to 3.2)	0.6 (0.3 to 1.1)
	6 months n (intervention/control) = 199 /190	42 (21.1%)	14 (7.4%)	3.36 (1.77 to 6.4)^**^	3.4 (1.8 to 6.5)^*^	1.7 (0.9 to 3.2)	0.6 (0.3 to 1.1)
	12 months n (intervention/control) = 220/236	52 (23.6%)	30 (12.7%)	2.13 (1.30 to 3.5)^*^	2.2 (1.3 to 3.5)^*^	1.4 (0.8 to 2.4)	0.5 (0.3 to 0.8)^*^
**Use of PHQ-9**							
Pre	Baseline n (intervention/control) = 209/196	–	–	–	–	–	–
	3 months n (intervention/control) = 183/191	–	–	–	–	–	–
	6 months n (intervention/control) = 184/185	–	–	–	–	–	–
Post	Baseline n (intervention/control) = 202/190	9 (4.5%)	2 (2.2%)	4.38 (0.93 to 20.6)	4.2 (0.9 to 19.7)	0.3 (0 to 2.7)	0.6 (0.2 to 2.1)
	3 months n (intervention/control) = 199/190	13 (6.5%)	2 (1.1%)	6.57 (1.46 to 29.5)^*^	6.4 (1.4 to 29)^*^	0.9 (0.2 to 3.2)	0.6 (0.2 to 1.8)
	6 months n (intervention/control) = 199 /190	14 (7%)	2 (1.1%)	7.11 (1.59 to 31.7)^*^	7.1 (1.6 to 31.5)^*^	1.2 (0.4 to 3.9)	0.5 (0.2 to 1.5)
	12 months n (intervention/control) = 220/236	15 (6.8%)	1 (0.4%)	17.2 (2.25 to 131.3)^*^	17.3 (2.3 to 131.9)^*^	1.2 (0.4 to 3.8)	0.9 (0.3 to 2.5)

n = number of participants.

* *P* < 0.05.

** *P* ≤ 0.001.

The adjusted multivariable analysis revealed no significant differences in the clinical records of diagnosis of depression in the pre-implementation phase, except at baseline. In all phases of post-intervention follow-up, there were significant differences between the two groups, which persisted at 1 year after the intervention (OR = 1.93 (95% CI 1.29 to 2.88)). At 3 and 6 months, in the pre-implementation phase, the existence of physical comorbidities was significantly associated with the presence of depression in the clinical records. No such association was observed in the post-intervention sample.

The intervention produced a notable impact on the stratification of the severity of depression. Thus, during the post-implementation phase, the PCPs in the intervention group differentiated the level of depression (as mild, moderate or severe) to a much greater degree than before. This significant change did not take place in the control group. However, in the analysis adjusted by gender and physical comorbidity, this difference was only noted in the third month post-implementation.

Regarding the impact of the intervention on the use of structured methods (i.e., PHQ-9), in the pre-implementation phase, this instrument was very little used. Significant differences in this respect were observed in the crude analysis of the post-implementation phase. In the adjusted analysis, although the number of PHQ-9 records remained limited, significant differences in favour of the intervention group appeared after 3 months, and this increased use persisted at 12 months.

Multilevel logistic regression to evaluate the “healthcare centre” effect on the incidence of records of depression in the clinical records showed a minimal variation among clusters, with ICCs that did not exceed 3% and equal MOR in the “pre” phase. In the “post” phase, similar findings were obtained, with ICC values below 5% and MOR values that did not show any change in the probability of change in the diagnosis of depression in clinical records attributable to the health centre. In the same way, regarding the record of the level of severity of depression, no significant differences were obtained in the multilevel analysis. For PHQ-9 utilisation, during the post phase, an increase in variability was observed attributable to the “healthcare centre” variable in those centres belonging to the intervention group, which increased by more than double the probability of using the PHQ-9 at 12 months, with a ICC value of 15.9% and a MOR value of 2.55.

## Discussion

The overall aim of this study was to evaluate the impact of the implementation of clinical guidelines for the management of depression in primary health care through a multicomponent provider-centred intervention, based on three parameters: the detection of depressive disorders by PCPs; the identification of the severity of depression and the use of a standardised instrument (PHQ-9) to support the diagnosis.

Reflecting the quasi-experimental nature of the study, the first phase required us to determine whether there were differences between the health centres in the prevalence of depression in the intervention and control groups. The prevalence values found were slightly lower than those reported in previous studies of similar populations in the primary healthcare context in Spain (Caballero *et al.*, 2008; Gabilondo *et al.*, 2010). Importantly, our findings confirm that there were no prior disparities in the epidemiological distribution of depressive processes due to social inequalities (Araya *et al.*, 2018; Patel *et al.*, 2018). Moreover, prevalence values obtained in studies conducted in other countries are comparable with those obtained in our study, while higher values have been reported when DSM-V criteria are applied (Hasin *et al.*, 2018).

With regard to the impact of the implementation of the clinical guidelines on the PCPs’ usual practice, the differences found in terms of gender, for the pre-implementation phase, may be attributable to the frequent imbalances in some variables produced by the randomisation process (Lin *et al.*, 2015). Since depression is more common among women than men, the differences observed suggest that these data could influence the diagnosis of depression. However, the results obtained reflect the opposite, as there was no influence of this difference on the results during the pre-implementation phase. Moreover, these differences disappeared in the post-implementation phase, and therefore, it is very unlikely that the gender balance influenced the results.

Concerning the presence of the diagnosis of depression in the clinical records, the values obtained for this variable, after implementation of the clinical guidelines, increased to a significantly greater degree in the intervention group versus the control group. The OR of detection at 6 months after the implementation was similar to that reported by Sinnema *et al.* (2015) (1.68 versus 1.60, respectively). Additionally, our study extended follow-up to 12 months, revealing long-term persistence in the effect produced by the intervention on the recognition of depression. After implementation, the association between recording of depression and the presence of depression was attenuated. One of the reasons could be that when patients present associated physical diseases, the recording of the severity of depression is reduced, because it is very likely that PCPs focus their attention on the physical complaints.

Before the implementation of the clinical guidelines, the PCPs made minimal use of PHQ-9. In the intervention group, the use of this instrument increased, albeit to a limited degree. According to the barrier analysis performed, the PCPs reported that PHQ-9 was unlikely to be used if it was not available in the electronic medical records (Nogueras *et al.*, 2017). This instrument has proven to be a fast, easy method to support the diagnosis of depression. Moreover, the use of standardised instruments such as this, which address the main depressive symptoms and not only the somatic ones (such as loss of appetite or sleep disturbance), invites the practitioner to explore more emotional, motivational aspects during the assessment and even to consider the impact on the person’s daily life, thus converting the traditional biomedical assessment into a biopsychosocial one. Furthermore, the use of this or similar instruments makes it possible for the practitioner to categorise the severity of the patient’s depression in a relatively simple way (Henry *et al.*, 2020). This question is of essential importance, since the intervention adopted will depend, in every case, on the severity detected (García-Herrera Pérez-Bryan *et al.*, 2011). These instruments have also proven useful in assessing the long-term evolution of depression, facilitating follow-up of the patient’s condition (Muñoz-Navarro *et al.*, 2017; Wikberg *et al.*, 2017), especially when used in conjunction with instruments that are more specifically aimed at measuring change, such as the Global Rating of Change (Robinson *et al.*, 2017). These characteristics are very useful in primary health care, where most depressive disorders are detected. However, as we show, if PHQ-9 is not available in the electronic medical records, it is much less likely to be used by the PCP. In fact, some PCPs within the control group started using PHQ-9 after the implementation process, probably because the clinical guidelines were available in *GuiaSalud*, the National Resource Directory available to all PCPs, where they can consult clinical recommendations and where physicians can share useful information.

This study was designed taking into account previous work in this field (Aakhus *et al.*, 2016; Flodgren *et al.*, 2016). Specifically, our analysis of barriers and facilitators during the intervention is in line with other approaches described in the literature (Nogueras *et al.*, 2017; Richter-Sundberg *et al.*, 2015), such as barriers related to professionals, patients, organisational issues and the economic context. In our case, one of the most prominent barriers concerned the organisational and structural context, namely the lack of time and resources (Nogueras *et al.*, 2017). A relevant factor is that the project was carried out at a difficult moment in Spain, which was undergoing a major economic crisis that had provoked significant cuts in health services and staffing.

Among the limitations of this study, its design was only quasi-experimental, due to the impossibility of randomising the health centres (for organisational reasons). However, the selection of participants in each health centre was randomised, to minimise possible intragroup selection biases. Moreover, during the implementation phase it was not possible to obtain the participation of patients and their relatives as stakeholders, deploying interventions on their own behalf.

Likewise, the results of this study show an improvement in detection and diagnosis of depression, that is, on the healthcare process. However, it would be important for future studies to explore whether these changes in health care lead to an improvement in the mental health of user.

## Conclusion

A multicomponent intervention based on the TPB was carried out to enhance the detection and diagnosis of depression, via implementation of the corresponding clinical guidelines. This intervention significantly advanced the identification of the presence and severity of depression (as evidenced in the clinical records of primary care providers), although the presence of physical comorbidities reduced its impact.

## Supplementary material

For supplementary material accompanying this paper visit http://doi.org/10.1017/S1463423623000300. click here to view supplementary material
