# Supplementary material for: A multifaceted provider-centred intervention versus usual care to improve the recognition and diagnosis of depression in primary health care: a hybrid study
Source: Prim Health Care Res Dev. 2023 Jul 10;24:e45. doi: 10.1017/S1463423623000300 (PMC10372767; doi:10.1017/S1463423623000300)
Supplement: Supplementary file 1 [file S1463423623000300sup001.docx]

Supplementary file 1: Description of the ingredients of the interventions.

| **Selection of PCPs to lead the implementation** | |
| --- | --- |
| PCPs with a special interest in mental health were selected to lead the process of implementing the CG at their health centers, in coordination with the heads of each center, who had a thorough knowledge about the most appropriate profiles among the medical staff. | |
| **Presentation meeting with local providers** | |
| During this session, local PCPs were asked to select the most frequent areas of uncertainty when treating people with depression in their clinical practice. | |
| The training design for other PCPs was jointly configured between psychiatrists and PCPs. | |
| **Structure of the educational sessions for PCPs** | |
| Duration adapted to the availability and organizational context of each health center. | |
| Eight one-hour sessions aimed to identify the main issues in the management of people with depression were held in each health center. | |
| The workshops were leaded by PCPs with a special interest in mental health, with the support of a reference psychiatrist. | |
| All the training sessions were accredited by the Andalusian Agency for Health Care Quality | |
| Contents of the educational sessions: | Real life cases selected by PCPs. |
|  | Presentation of the case with the information usually available in daily practice. |
|  |  |
| Contextualization of the approach in clinical practice. | After the presentation of the case, the action plan with the patient presented was discussed with the participating PCP, including evaluation, diagnosis and treatment. |
|  | The decisions were contrasted with the recommendations of the CG.  Discussion of the best decisions and the barriers identified by PCPs. |
| The CG was provided in advance to participating primary care practitioners. | |
| Evaluation of pre-post test level of knowledge on topics about management of depression (evaluation, diagnosis and treatment). | |
| Last workshop aimed at detecting and discussing the barriers and facilitators.  The intervention lasted approximately 3 months in each health center. | |
| **Self Help Material** | |
| Self-help material for being shared with patients were designed and distributed with the CG.  Available at: <https://www.sspa.juntadeandalucia.es/servicioandaluzdesalud/el-sas/servicios-y-centros/salud-mental/guia-de-autoayuda-para-la-depresion-y-los-trastornos-de-ansiedad> | |

*PCP: primary care practitioners
